# *Streptococcus agalactiae* Sequence Type 283 in Farmed Fish, Brazil

**DOI:** 10.3201/eid2504.180543

**Published:** 2019-04

**Authors:** Carlos A.G. Leal, Guilherme A. Queiroz, Felipe L. Pereira, Guilherme C. Tavares, Henrique C.P. Figueiredo

**Affiliations:** Federal University of Minas Gerais, Belo Horizonte, Brazil

**Keywords:** Group B streptococcus, GBS, ST283, genome, wgMLST, clonal expansion, Nile tilapia, Streptococcus agalactiae, Brazil, bacteria, zoonoses, sequence type 283, farmed fish

## Abstract

In 2016 and 2017, we characterized outbreaks caused by *Streptococcus agalactiae* serotype III sequence type (ST) 283 in Nile tilapia farms in Brazil. Whole-genome multilocus sequence typing clustered the fish isolates together with the zoonotic ST283 and other STs related to cases in humans, frogs, dogs, cattle, and dolphins.

*Streptococcus agalactiae* (group B *Streptococcus* [GBS]) is a major etiologic agent of diseases in humans and animals (*1*). Episodes of bacteremia and meningitis in humans associated with raw fish consumption were reported in Singapore (*2**,**3*). Two case–control studies determined that GBS serotype III sequence type (ST) 283 was associated with disease in 9 (*2*) and 19 (*3*) patients in that country at different times during 2015. This specific ST has been identified as a zoonotic agent to humans and already has been detected in freshwater fish dishes from food stalls in Singapore (*1*); consumption of such fish dishes led to increased cases during that year. In farmed fish, GBS serotype III ST283 was detected in diseased tilapia (*Oreochromis* sp.) in Thailand (*4*).

In Brazil, fish-pathogenic GBS has been isolated mainly from farmed Nile tilapia (*Oreochromis niloticus*) since the 2000s. The predominant serotype in Brazil is serotype Ib from ST260, ST927, and a nontypeable ST; all these STs are reported as fish-adapted pathogens (*5*). Sporadic detection of serotype Ia ST103 also was described in Brazil (*6*). However, since 2016, a new serotype, serotype III, has emerged in the country. This serotype has been detected in different Nile tilapia farms in the northeastern region (*7*). The genetic diversity and ST of these isolates have not yet been identified.

Our objective was to evaluate, by molecular and genomic approaches, the GBS serotype III isolates from outbreaks in Nile tilapia farms in Brazil. We also aimed to study the genetic relationship between isolates from farmed fish in Brazil, from foodborne isolates from humans in Asia, and from cases in fish.

## The Study

**Figure 1:**
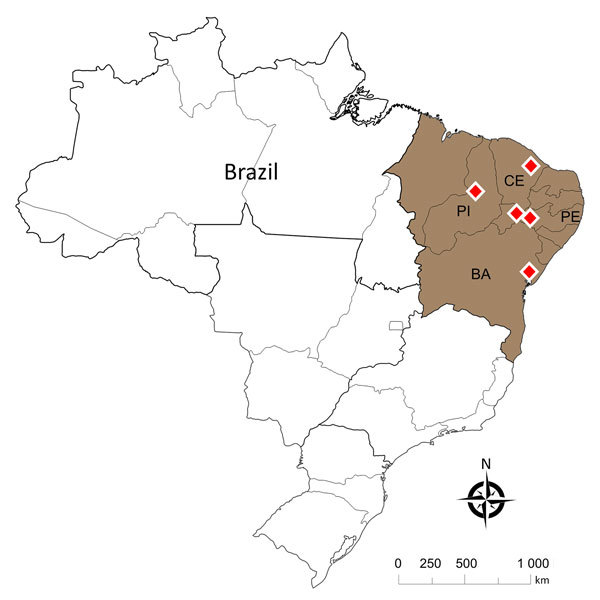
Location of the deaths of farmed Nile tilapia (*Oreochromis niloticus*) (shaded area) caused by *Streptococcus agalactiae* serotype III sequence type 283 (red diamonds), Brazil. BA, Bahia state; CE, Ceará state; PE, Pernambuco state; PI, Piauí state; ST, sequence type.

During July 2016–June of 2017, we followed 6 outbreaks that led to Nile tilapia deaths at commercial farms in 4 Brazilian states: Piauí, Pernambuco, Ceará, and Bahia (Figure 1). Disease outbreaks with high death rates (daily rates >0.2% and total death rates in the herds of 25%–35%) in fish vaccinated with a commercial inactivated vaccine against GBS serotype Ib were reported. Diseased fish showed signs of lethargy, melanosis, and exophthalmia. We sampled 71 diseased fish and transported them on ice to the laboratory (Table), where we immediately performed bacteriologic analyses. Fish tissue sampling and the culture conditions of the analyses were as described previously (*8*). The isolates obtained from 64 fish were identified up to the species level by matrix-assisted laser desorption ionization time-of-flight mass spectrometry analysis, using a previously published method (*9*). We confirmed GBS as the principal pathogen associated with the deaths during all the outbreaks (Table).

**Table:** Characteristics of *Streptococcus agalactiae* outbreaks in Nile tilapia (*Oreochromis niloticus*), Brazil*

Outbreak	Date	State	No. fish tested	Diagnosis (no. fish)	Serotype	MLST
1	2016 Jul	Piauí	8	*S. agalactiae *(*7)*	III	283
2	2017 Mar	Pernambuco	21	*S. agalactiae (18)*	III	283
				*S. agalactiae *(*3*)	Ib	260
3	2017 Mar	Bahia	13	*S. agalactiae *(*12*),* S. dysgalactiae *(*1*)	III	283
4	2017 May	Ceará	7	*S. agalactiae *(*4*)	III	283
5	2017 May	Piauí	6	*S. agalactiae *(*2*)	III	283
				*S. dysgalactiae *(*1*)		
6	2017 Jun	Bahia	16	*S. agalactiae *(16)	III	283

*Capsular serotyping and MLST analyses were performed only for* S. agalactiae *isolates. MLST, multilocus sequence typing.

We performed capsular polysaccharide typing for all GBS isolates using a previously published multiplex PCR (*10*). In addition, we conducted sequencing of housekeeping genes for multilocus sequence typing (MLST) analysis (*11*). According to the GBS MLST database (http://pubmlst.org/sagalactiae), 59 isolates belonged to serotype III ST283 and 3 to serotype Ib ST260.

For selected GBS isolates from each farm, we performed a PCR-based fingerprinting technique (ERIC-PCR [enterobacterial repetitive intergenic consensus PCR]) described by Costa et al. (*12*) to evaluate genetic diversity in comparison with MLST results. We observed nondistinguishable banding patterns between all GBS serotype III ST283 isolates, regardless of their geographic origin (data not shown), suggesting the possibility of a genetically close relation.

To determine the genomic relationship between GBS serotype III ST283 isolates from fish in Brazil and Asia and foodborne cases in humans, we conducted whole-genome sequencing and whole-genome MLST (wgMLST) analyses. In brief, 8 isolates (SA01AQUAVET, SA06AQUAVET, SA12AQUAVET, SA22AQUAVET, SA90AQUAVET, SA98AQUAVET, SApx2AQUAVET, and SApx7AQUAVET), representing each outbreak case, and 3 isolates from outbreak 2, because the isolates of outbreak 2 were obtained from 3 different farms, were selected (Appendix).

**Figure 2:**
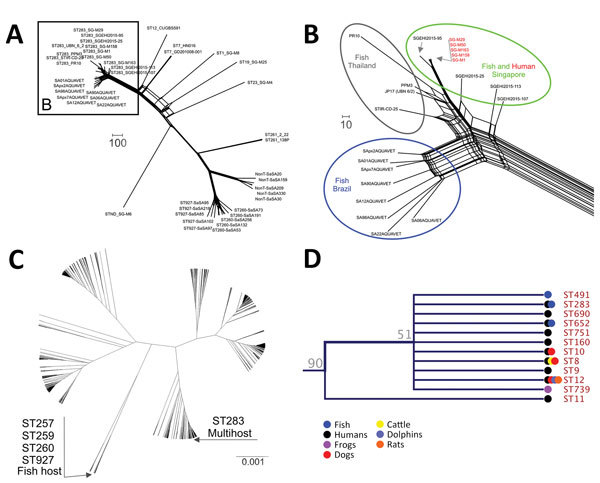
Phylogenetic analyses of *Streptococcus agalactiae* strains. A) Phylogenomic neighbor network of whole-genome multilocus sequence typing data of 45 group B *Streptococcus* (GBS) strains. Scale bar measures 10 different alleles between the isolates. B) Magnified image from panel A showing GBS ST283 phylogenomic splits. Isolates obtained from clinical cases of diseased fish in Brazil (blue circle) and Thailand (gray circle) and isolates from foodborne outbreaks in Singapore (green circle). Scale bar measures 100 different alleles between the isolates. C) Phylogenetic relationship of all GBS STs concatenated using CLC Genomics Workbench (QIAGEN, https://www.qiagen.com) and generated using UPGMA (unweighted pair group method with arithmetic mean). GBS strains isolated exclusively from fish are genetically distant from ST283 cluster. Scale bar measures nucleotide substitutions per site. D) ST283 clade comprises multihost strains. ST, sequence type.

In the wgMLST analysis, GBS serotype III ST283 isolates formed 1 phylogenomic related group (Figure 2, panel A). We compared 3,539 loci of the analyzed GBS isolates based on pairwise comparisons of the numbers of homologous loci with distinct allele sequences and found that all ST283 isolates (from fish and humans) displayed up to 505 distinct allele sequences. When we compared isolates from Brazil with fish strains from Asia, we observed variations ranging from 136 to 505 (3.84%–14.26%) (Figure 2, panel B) in loci. Among isolates from humans and fish in Asia, the variations were 31–240 (0.87%–6.78%), whereas for isolates from humans in Asia compared with from fish in Brazil, the variations were 272–415 (7.68%–11.72%). GBS ST283 from Brazil showed loci variations ranging from 71 to 256 between each other. These levels of local diversities within an ST were reported in a previous study that evaluated distinct STs obtained from fish in Brazil (*5*).

Finally, we conducted a phylogenetic analysis with MLST loci concatenated (Figure 2, panels C, D). The 7 loci (i.e., adhP, pheS, atr, glnA, sdhA, glcK, and tkt) of each of 1,193 STs, available in GBS MLST database) were concatenated and aligned using CLC Genomics Workbench (QIAGEN, https://www.qiagen.com). From the alignment results, we generated a phylogenetic tree in the CLC Genomics Workbench using the UPGMA (unweighted pair group method with arithmetic mean) construction method, Jukes-Cantor as the nucleotide distance measure, and 1,000 replicates on bootstrap analysis. ST283 formed a consistent (bootstrap 90%) clade with other STs with different hosts other than fish (Figure 2, panel D), such as humans, fish, and frogs, including some with multihost range, among them humans, fish, cattle, dolphins, dogs, and frogs.

## Conclusions

We described the emergence of GBS ST (GBS serotype III ST283) associated with the Nile tilapia infection in Brazil. ST283 had been previously detected in fish (with and without clinical disease) only in Southeast Asia countries (*1*,*4*). This report in Brazil indicates possible spread of this pathogen genotype around the world. Based on import records of live Nile tilapia from Singapore to Brazil in 2014 and our analyses (ERIC-PCR, MLST, and wgMLST), it is possible that this genotype was introduced into the country with the recently imported fish.

GBS serotype III ST283 caused severe foodborne outbreaks in Singapore in 2015, and these outbreaks have been linked to raw fish consumption (*2*), indicating a zoonotic lineage for this serotype. Our results demonstrate that GBS serotype III ST283 isolates from Brazil clustered in the same network branch with GBS isolates from Asia, from fish and human hosts; however, we observed genomic diversity that depicted a clonal expansion of this genotype. The genomic diversity among GBS isolates from human and fish hosts was also observed in a previous study (*1*), which further reinforces the concept of a lineage that is adapted to >1 host. This previous study on GBS suggested that ST283 might not be pathogenic for fish (*1*). However, a previous study (*4*) found that it was associated with disease in fish. After evaluating isolates of tilapia clinical disease cases, we confirmed that this ST283 is associated with multiple outbreaks in commercial tilapia farms. Thus, it demonstrates the clear pathogenic behavior of this ST to fish. Moreover, genomic analysis indicates it belongs to a cluster with other STs from multiple susceptible hosts (Figure 2, panel D), such as the ST12, which was isolated from humans, dogs, dolphins, and rats, corroborating the suggestion that the member of this clade affects multiple host species. ST283 was only isolated from diseased tilapia, which cannot be used for human consumption. Future longitudinal noncasuistic study is needed to identify the occurrence and prevalence of GBS infection by the ST283 lineage also in asymptomatic fish before and after the outbreaks. In this context, asymptomatic tilapia carriers might represent a serious public health concern.

Appendix Additional methods for study of *Streptococcus agalactiae* sequence type 283 in farmed fish, Brazil.
