# Supplementary material for: Streptococcus agalactiae Sequence Type 283 in Farmed Fish, Brazil
Source: Emerg Infect Dis. 2019 Apr;25(4):776–9. doi: 10.3201/eid2504.180543 (PMC6433023; doi:10.3201/eid2504.180543)
Supplement: Appendix — Additional methods for study of Streptococcus agalactiae sequence type 283 in farmed fish, Brazil. [file 18-0543-Techapp-s1.pdf]

Article DOI: https://doi.org/10.3201/eid2504.180543

Streptococcus agalactiae Sequence Type 283 in Farmed Fish, Brazil

Appendix

Additional Methods

Sequencing and Assembly

The isolates SA01AQUAVET, SA06AQUAVET, SA12AQUAVET, SA22AQUAVET, SA90AQUAVET, SA98AQUAVET, SApx2AQUAVET ,and SApx7AQUAVET had their genomes sequenced by Ion Torrent Personal Genome Machine (PGM) using the Ion PGM Sequencing 400bp kit, according to the manufacturer's instructions. An in-house script (https://www.github.com/aquacen/fast_sample) was used to obtain reads with a PHRED quality score $\leq$20 (i.e., –q 20 parameter) and to exclude adaptor sequences (i.e., –l 17 parameter). Genome sequences were then de novo assembled using SPAdes v3.9.1 (*1*) with parameters "–iontorrent and -k15,21,35,55,99,127."

Whole-genome Multilocus Sequence Typing

The draft genome sequences of the aforementioned isolates and additional genome sequences (n = 37) of isolates from different genotypes and hosts (Appendix Table), including genomes from clinical cases of streptococcosis in humans (associated with raw fish consumption in Southeast Asia) were submitted to phylogenomic analysis based on whole genome MLST. The genomes were submitted to Bacterial Isolate Genome Sequence Database (BIGSdb), where all loci were compared 1 by 1 between isolates using a gene-by-gene approach (*2*) in a GenomeComparator plugin. All loci scheme was previously generated using all gene clusters from cd-hit-est software v4.6 (*3*) with file containing predicted genes from all strains and default parameters. Gene prediction was performed using Prokka 1.11 (*4*) with default parameters. A distance matrix with the relative genomic divergence between all isolates was obtained and subsequently used to construct a phylogenomic NeighborNet network using SplitsTree 4.0 (*5*).

References

1. Bankevich A, Nurk S, Antipov D, Gurevich AA, Dvorkin M, Kulikov AS, et al. SPAdes: a new genome assembly algorithm and its applications to single-cell sequencing. J Comput Biol. 2012;19:455–77. PubMed http://dx.doi.org/10.1089/cmb.2012.0021

2. Maiden MCJ, Jansen van Rensburg MJ, Bray JE, Earle SG, Ford SA, Jolley KA, et al. MLST revisited: the gene-by-gene approach to bacterial genomics. Nat Rev Microbiol. 2013;11:728–36. PubMed http://dx.doi.org/10.1038/nrmicro3093

3. Li W, Godzik A. Cd-hit: a fast program for clustering and comparing large sets of protein or nucleotide sequences. Bioinformatics. 2006;22:1658–9. PubMed http://dx.doi.org/10.1093/bioinformatics/btl158

4. Seemann T. Prokka: rapid prokaryotic genome annotation. Bioinformatics. 2014;30:2068–9. PubMed http://dx.doi.org/10.1093/bioinformatics/btu153

5. Huson DH, Bryant D. Application of phylogenetic networks in evolutionary studies. Mol Biol Evol. 2006;23:254–67. PubMed http://dx.doi.org/10.1093/molbev/msj030

6. Delannoy CM, Crumlish M, Fontaine MC, Pollock J, Foster G, Dagleish MP, et al. Human *Streptococcus agalactiae* strains in aquatic mammals and fish. BMC Microbiol. 2013;13:41. PubMed http://dx.doi.org/10.1186/1471-2180-13-41

7. Areechon N, Kannika K, Hirono I, Kondo H, Unajak S. Draft genome sequences of *Streptococcus agalactiae* serotype Ia and III isolates from tilapia farms in Thailand. Genome Announc. 2016;4:e00122–16. PubMed http://dx.doi.org/10.1128/genomeA.00122-16

8. Chau ML, Chen SL, Yap M, Hartantyo SHP, Chiew PKT, Fernandez CJ, et al. Group B streptococcus infections caused by improper sourcing and handling of fish for raw consumption, Singapore, 2015–2016. Emerg Infect Dis. 2017;23:2002. PubMed http://dx.doi.org/10.3201/eid2312.170596

9. Kalimuddin S, Chen SL, Lim CTK, Koh TH, Tan TY, Kam M, et al.; Singapore Group B Streptococcus Consortium. 2015 Epidemic of severe *Streptococcus agalactiae* sequence type 283 infections in Singapore associated with the consumption of raw freshwater fish: a detailed analysis of clinical, epidemiological, and bacterial sequencing data. Clin Infect Dis. 2017;64(suppl_2):S145–52. PubMed http://dx.doi.org/10.1093/cid/cix021

10. Wang R, Li L, Huang Y, Luo F, Liang W, Gan X, et al. Comparative genome analysis identifies two large deletions in the genome of highly-passaged attenuated *Streptococcus agalactiae* strain YM001 compared to the parental pathogenic strain HN016. BMC Genomics. 2015;16:897. PubMed http://dx.doi.org/10.1186/s12864-015-2026-y

11. Liu G, Zhang W, Lu C. Complete genome sequence of *Streptococcus agalactiae* GD201008-001, isolated in China from tilapia with meningoencephalitis. J Bacteriol. 2012;194:6653. PubMed http://dx.doi.org/10.1128/JB.01788-12

12. Barony GM, Tavares GC, Pereira FL, Carvalho AF, Dorella FA, Leal CAG, et al. Large-scale genomic analyses reveal the population structure and evolutionary trends of *Streptococcus agalactiae* strains in Brazilian fish farms. Sci Rep. 2017;7:13538. PubMed http://dx.doi.org/10.1038/s41598-017-13228-z

13. Rosinski-Chupin I, Sauvage E, Mairey B, Mangenot S, Ma L, Da Cunha V, et al. Reductive evolution in *Streptococcus agalactiae* and the emergence of a host adapted lineage. BMC Genomics. 2013;14:252. PubMed http://dx.doi.org/10.1186/1471-2164-14-252

14. Pridgeon JW, Zhang D. Complete genome sequence of a virulent *Streptococcus agalactiae* strain, 138P, isolated from diseased Nile tilapia. Genome Announc. 2014;2:e00295–14. PubMed

Appendix Table. Isolates used in wgMLST analysis*

ID no.	Country	Host	ST	GenBank accession no.	Reference
SA01AQUAVET	Brazil	Fish	283	PVLI00000000	This study
SA06AQUAVET	Brazil	Fish	283	PVLJ00000000	This study
SA12AQUAVET	Brazil	Fish	283	PVLK00000000	This study
SA22AQUAVET	Brazil	Fish	283	PVLL00000000	This study
SA90AQUAVET	Brazil	Fish	283	PVLM00000000	This study
SA98AQUAVET	Brazil	Fish	283	PVLN00000000	This study
SApx2AQUAVET	Brazil	Fish	283	PVLO00000000	This study
SApx7AQUAVET	Brazil	Fish	283	PVLP00000000	This study
STIR-CD-25	Thailand	Fish	283	NZ_ANEK00000000	(*6*)
JP17 (UBN 6/2)	Thailand	Fish	283	NZ_BCNJ00000000	(*7*)
PPM3	Thailand	Fish	283	PTJJ00000000	NCBI database
PR10	Thailand	Fish	283	PTJK00000000	NCBI database
SGEHI2015–107	Singapore	Fish	283	CP025027.1	(*8*)
SGEHI2015–113	Singapore	Fish	283	CP025026.1	(*8*)
SGEHI2015–25	Singapore	Fish	283	CP025029.1	(*8*)
SGEHI2015–95	Singapore	Fish	283	CP025028.1	(*8*)
SG-M1	Singapore	Human	283	CP012419.2	(*9*)
SG-M158	Singapore	Human	283	CP021864.1	(*9*)
SG-M163	Singapore	Human	283	CP021863.1	(*9*)
SG-M29	Singapore	Human	283	CP021866.1	(*9*)
SG-M50	Singapore	Human	283	CP021865.1	(*9*)
SG-M8	Singapore	Human	1	CP021868.1	(*9*)
HN016	China	Fish	7	CP011325.1	(*10*)
GD201008–001	China	Fish	7	CP003810.1	(*11*)
CUGBS591	Hong Kong	Human	12	CP021862.1	(*9*)
SG-M25	Singapore	Human	19	CP021867.1	(*9*)
SG-M4	Singapore	Human	23	CP021870.1	(*9*)
SA53	Brazil	Fish	260	CP019802.1	(*12*)
SA73	Brazil	Fish	260	CP019803.1	(*12*)
SA132	Brazil	Fish	260	CP019815.1	(*12*)
SA191	Brazil	Fish	260	CP019819.1	(*12*)
SA256	Brazil	Fish	260	CP019827.1	(*12*)
2–22	Israel	Fish	261	FO393392.1	(*13*)
138P	USA	Fish	261	CP007482.1	(*14*)
SA85	Brazil	Fish	927	CP019811.1	(*12*)
SA95	Brazil	Fish	927	CP019812.1	(*12*)
SA97	Brazil	Fish	927	CP019813.1	(*12*)
SA102	Brazil	Fish	927	CP019814.1	(*12*)
SA218	Brazil	Fish	927	CP019824.1	(*12*)
SA20	Brazil	Fish	NT†	CP003919.2	(*12*)
SA30	Brazil	Fish	NT	CP019800.1	(*12*)
SA159	Brazil	Fish	NT	CP019817.1	(*12*)
SA209	Brazil	Fish	NT	CP019822.1	(*12*)
SA330	Brazil	Fish	NT	CP019829.1	(*12*)
SG-M6	Singapore	Human	ND‡	CP021869.1	(*9*)

*ID, identification; ST, sequence type; wgMLST, whole-genome multilocus sequence typing.
†Nontypeable.
‡Nondetermined.
